# The discovery of a catalytic RNA within RNase P and its legacy

**DOI:** 10.1016/j.jbc.2024.107318

**Published:** 2024-04-25

**Authors:** Leif A. Kirsebom, Fenyong Liu, William H. McClain

**Affiliations:** 1 Department of Cell and Molecular Biology, Uppsala University, Uppsala, Sweden; 2 School of Public Health, University of California, Berkeley, California, USA; 3 Department of Bacteriology, University of Wisconsin-Madison, Madison, Wisconsin, USA

**Keywords:** RNase P, catalytic RNA, RNase P processing, RNase P and metal ions, RNase P and application, RNA biology, RNA world, RNA structure

## Abstract

Sidney Altman's discovery of the processing of one RNA by another RNA that acts like an enzyme was revolutionary in biology and the basis for his sharing the 1989 Nobel Prize in Chemistry with Thomas Cech. These breakthrough findings support the key role of RNA in molecular evolution, where replicating RNAs (and similar chemical derivatives) either with or without peptides functioned in protocells during the early stages of life on Earth, an era referred to as the RNA world. Here, we cover the historical background highlighting the work of Altman and his colleagues and the subsequent efforts of other researchers to understand the biological function of RNase P and its catalytic RNA subunit and to employ it as a tool to downregulate gene expression. We primarily discuss bacterial RNase P–related studies but acknowledge that many groups have significantly contributed to our understanding of archaeal and eukaryotic RNase P, as reviewed in this special issue and elsewhere.

Francis Crick's note to the RNA Tie Club titled "On degenerate templates and the adaptor hypothesis" introduced the existence of an adaptor molecule that connects the gene sequence with the protein sequence [(http://resource.nlm.nih.gov/101584582X73) (1); the informal scientific RNA Tie Club was founded 1954 for scientists with an interest in protein synthesis and the genetic code (2)]. A few years later, a small adaptor molecule (about 75 nt, in length) termed pH 5 RNA or soluble RNA was identified biochemically. The molecule's sedimentation coefficient was determined to be 4S, and in 1960, it was renamed tRNA (3, 4, 5). Subsequently, sequencing of a yeast alanine tRNA revealed a 76 residue-long polynucleotide chain (6).

To pursue the understanding of tRNA structure and function, Sidney Altman began his postdoctoral work under the mentorship of Sydney Brenner and Francis Crick at the Medical Research Council Laboratory of Molecular Biology (LMB) in Cambridge, England. John Smith was recruited to the LMB for his expertise in the primary structure determinations of RNAs, and he headed the new RNA biochemistry subsection under Brenner and Crick. Two of Altman's postdoctoral colleagues were Bill McClain and Hugh Robertson, who brought expertise in bacteriophage genetics and ribonuclease purification, respectively. The amber UAG triplet is nonsense in mRNA (*i.e*., does not correspond to an amino acid codon) and terminates protein synthesis in normal cells. However, the amber UAG triplet is translated in a cell having a nonsense suppressor tRNA that inserts a specific amino acid, for example, tyrosine (tRNA^Tyr^Su3). Brenner and his laboratory developed the tactics for applying genetic selections, first by providing an active amber-suppressor tRNA^Tyr^Su3 gene carried by bacteriophage φ80 and then by isolating derivative strains with diminished suppressor tRNA activity or with an altered amino acid acceptor specificity. The first phase established that suppression resulted from a nucleotide change in the tRNA anticodon (7). At the project's outset, the notion was that a mutated tRNA would exhibit diminished function and appear as a less active suppressor tRNA when assayed (8). Using a polyacrylamide gel–based analysis of *in vivo* ^32^P-labeled RNAs, Altman reported the absence of mutant tRNA^Tyr^Su3 and the presence of a new RNA that was susceptible to cellular degradation and migrated more slowly in gels than "WT" tRNA^Tyr^Su3 (9).

To Altman's (and science's) lasting benefit, base-change mutations in tRNA perturbed molecular folding and thus the kinetics with which RNA-processing enzymes handle mutant tRNA precursor intermediates. Consequently, these intermediates accumulate, spending more time in incomplete maturation states. Upon subjecting this new RNA species to ribonuclease T_1_ digestion, Altman *et al*. estimated that it contains "approximately forty more nt than the usual tyrosine tRNA". RNA sequence determination confirmed the molecule as a tRNA^Tyr^Su3 precursor with a 5′-pppG remaining from transcription initiation and containing 41 extra nt in the 5′ precursor segment plus three additional nt in the 3' segment of the mature tRNA^Tyr^Su3 [Fig. 1*A* (10); it was later shown that the 5′ leader is 43 nt long]. The precursor did not contain modifications. The detection of a nucleolytic activity (later named RNase P) in the extracts of *Escherichia coli* producing tRNA^Tyr^Su3 from its precursor RNA announced the entry of RNase P into the biogenesis of mature tRNAs. The large "Altman RNA molecule" represented the first sequence of a tRNA precursor and reflected the general nature of and structure of tRNA precursor transcripts. Previous work had reported on large unstable transcripts in eukaryotes, possibly tRNA precursors, but these molecules were not sequenced (11, 12, 13). In conclusion, the combined results indicated that tRNA genes in both bacteria and mammals are transcribed as longer precursor RNAs, pre-tRNAs, which are enzymatically converted into mature tRNAs. Evidence soon emerged for parallels even with phage-encoded tRNAs.

**Figure 1:**
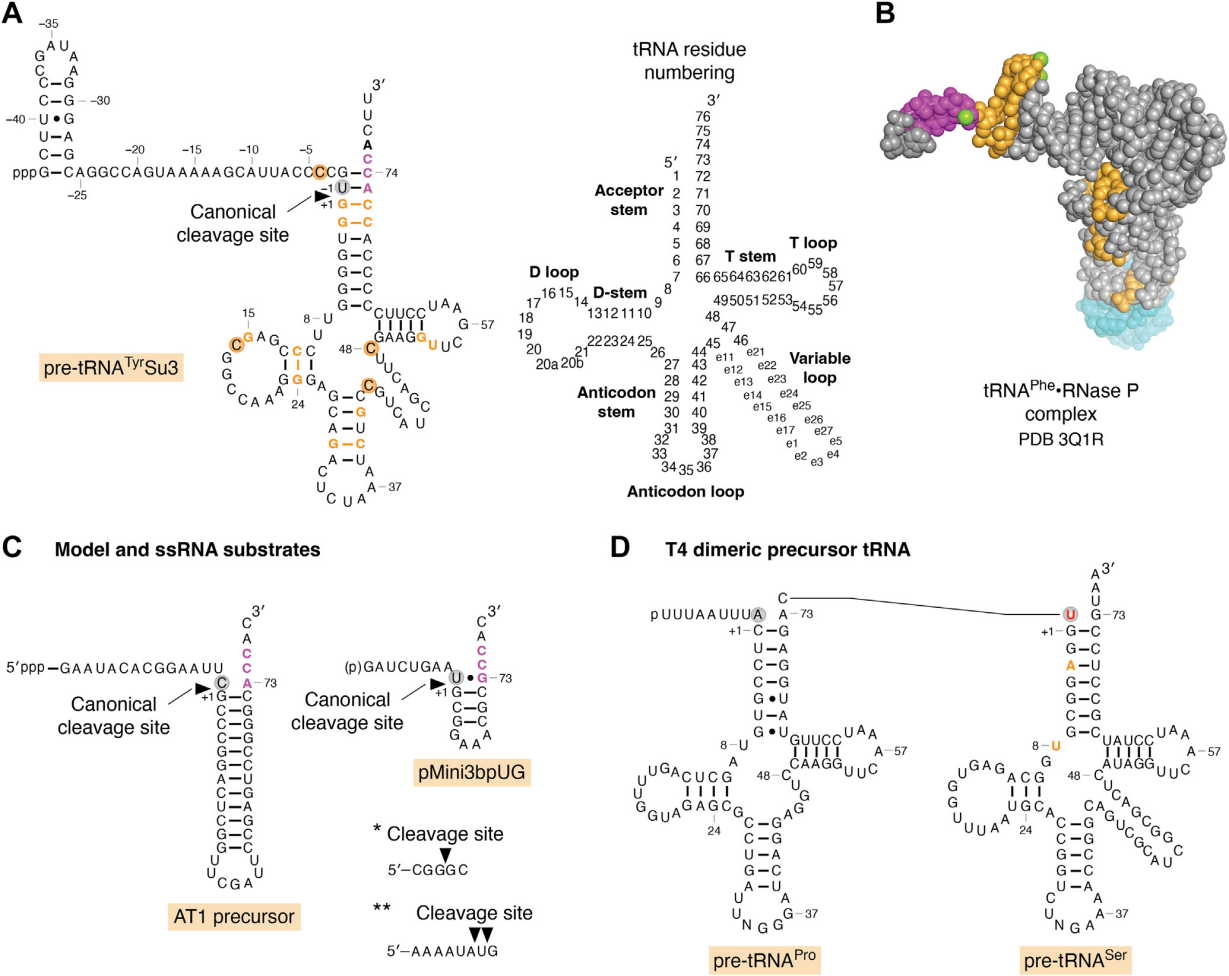
**Structures of monomeric, dimeric, and model RNase P substrates.***A*, residues in pre-tRNA^Tyr^Su3 implicated or demonstrated to be important in RNase P processing *in vivo* and/or *in vitro* are marked in *orange* and *magenta* (residues at the 3′ end that bp with the 5′GGU sequence in the RPR forming the "RCCA-RPR interaction"; Fig. 2*C*), while U_-1_ is highlighted with a *gray* circle. With respect to changes in pre-tRNA^Tyr^Su3 at the positions marked in *orange* and *magenta*, see the main text for details (9, 20, 44, 45, 85, 86, 269). Numbering of tRNA residues according to Steinberg *et al.* (270). *B*, the crystal structure of matured tRNA^Phe^ bound to RNase P [pdb 3Q1R (33)]. The color code for some pre-tRNA^Su3^Tyr residues (in *orange*, G_+1_, G_+2_, U_+8_, C_+11_, G_+15_, G_+24_, G_+30_, C_+40_, C_+71_, and C_+72_; in *magenta*, A_+73_, C_+74_, and C_+75_) shown to affect RNase P cleavage is the same as in panel A except U_+8_, but see panel E, T4 tRNA^Ser^. The *green spheres* represent Me(II)-ions seen in the RNase P-tRNA crystal structure. The *turquoise spheres* correspond to extra residues (not originally present in tRNA), which were added to promote crystallization (33). *C*, structures of model and ssRNA substrates. i) The AT1 precursor is derived by "combining" the acceptor-stem, the T-stem, and the T-loop of *Escherichia coli* tRNA^Phe^ (64), and the pMini3bpUG is derived from the *E. coli* pre-tRNA^Ser^Su1 precursor (98). Residues marked in *magenta* in both substrates interact with the 5′ GGU in the RPR, while C_-1_ and U_-1_ are highlighted with *gray* circles. The two short ssRNA substrates are cleaved by ∗*Bsu* RNase P (holoenzyme) and *Bsu* RPR/*Eco* C5 protein [reconstituted holoenzyme (70)] and ∗∗*Bsu* RNase P [holoenzyme (69)]. The cleavage sites are marked with *solid arrows*. *D*, the dimeric T4 Pro-Ser tRNA precursor. Residues influencing RNase P processing *in vivo* and *in vitro*, A_+2_ and U_+8_, are marked in *orange* (21). The *red*-colored U corresponds to pre-tRNA^Ser^ U_-1_.

McClain's interest in RNA sequencing led him to focus on the small RNAs of bacteriophage T4 that turned out to be tRNAs. Finding two RNAs with sizes longer than those expected of tRNAs seemed outside his interest. However, Robertson suggested incubating the longer-sized RNAs with his "30,000*g* supernatant fraction of *E. coli* MRE600 …," which contained RNase P but was otherwise relatively free of RNases. The incubation of one of the large T4 RNAs produced two smaller RNA products (later identified as tRNA^Pro^ and tRNA^Ser^, each with a 5′ end of the corresponding mature tRNA), while the second produced two smaller RNA products, tRNA^Thr^ and tRNA^Ile^ (14). Altman and Smith (10) had shown that the same extract also cleaved the precursor tRNA^Tyr^Su3 to generate mature tRNA^Tyr^Su3. Because neither of the larger T4 bands contained a 5′-pppG, neither is a primary transcript. Thus, these analyses concluded that a tRNA precursor could have the sequences of two tandem tRNAs, then a novelty in tRNA biology. The processing of these pre-tRNAs were later shown to depend on the *E. coli* host enzymes, BN nuclease, tRNA nucleotidyltransferase, and RNase P, to generate tRNAs with matured 5′ and 3′ termini (15, 16).

An endoribonuclease from the *E. coli* 30,000*g* supernatant fraction (see above) was purified and demonstrated to be responsible for removing the 5′ leader (Fig. 1*A*), and the ribonuclease was named RNase P (17). At the same time, temperature-sensitive (*ts*) *E. coli* mutants defective in generating functional tRNA^Tyr^Su3 nonsense suppressors were isolated by Schedl and Primakoff and by Shimura and Ozeki (18, 19). Genetic mapping identified at least two genes associated with RNase P activity, and these genes were later shown to encode for the RNase P protein (in *E. coli*, the subunit is called C5 protein; see below) and the other M1 RNA [for reviews of the early works (20, 21)].

The Altman laboratory continued the work on RNase P and discovered that M1 RNA is essential for *E. coli* RNase P activity. In 1981, Kole and Altman stated, "The absence of any demonstrable hydrolytic activity of the C5 protein is striking and implies that the M1 RNA must be involved in activating the catalytic mechanism of the RNase P complex" (22). The subsequent reconstitution of RNase P activity with purified M1 RNA and C5 protein allowed this conclusion: "The catalytic activity resides in M1 RNA." (23). Guerrier-Takada and Altman conclusively showed that this is indeed the case using M1 RNA produced by *in vitro* SP6 RNA polymerase transcription (24). With pure RNA in hand, the Altman laboratory continued to experimentally tackle the radical concept that an RNA, the M1 RNA, acts as a true trans-acting multiple-turnover catalyst that follows Michaelis–Menten kinetics with substrate binding, cleavage, and product release. For activity, RNase P requires Mg^2+^, and in the RNA-alone reaction, a higher Mg^2+^ concentration was needed than in the presence of the C5 protein (23, 24, 25). All of the scientific community did not embrace the discovery of M1 RNA as a bona fide catalyst. At about the same time, however, Cech et al. were identifying and characterizing a self-splicing RNA, the group I intron. The Cech group coined the term ribozyme to distinguish RNA catalysts from protein enzymes (26). Whereas the group I intron RNA acted in *cis* within the same RNA molecules, M1 RNA is a *trans*-acting ribozyme allowing multiple turnover.

## Understanding of an RNA catalyst: Making sense of the unexpected

The breakthrough discoveries that RNA can act as a catalyst inspired increased research in RNA biology [and the "RNA world" (27, 28)], including RNase P. Several laboratories and research groups pursued efforts to unravel the function of RNase P and its subunits by studying bacterial, archaeal, and eukaryotic RNase P. In contrast to bacterial RNase P, archaeal and eukaryotic RNase P are composed of 4 to 5 and 9 to 10 proteins, respectively, and one RNA (29). Despite these differences in protein composition, the RNA has been demonstrated to be the catalytic subunit in all domains of life (30, 31, 32). The crystal and cryo-EM structures of bacterial, archaeal, and eukaryotic RNase P in complex with tRNA or pre-tRNA have been reported (33, 34, 35, 36, 37) and advanced our understanding of this catalytic RNA.

Here, we focus on bacterial RNase P and survey findings that have contributed to our current understanding of the function of RNase P and its subunits by discussing the following: (i) pre-tRNA recognition and cleavage site selection by RNase P, (ii) RNase P RNA regions that interact with the substrate and the RNase P protein, (iii) importance of residues and chemical groups at and near the RNase P cleavage site, (iv) metal(II)-ions and the current understanding of the cleavage mechanism, and (v) some future challenges. Following this, we will discuss the use of RNase P and its catalytic RNA as pharmaceutical and biotechnology tools. We refer to RNase P RNA as RPR and the protein subunit as RPP (in *E. coli* or *Eco*, they are referred to as M1 RNA and the C5 protein, respectively). Residues in the pre-tRNA 5′ leader are referred as N_-1_, N_-2_ etc, where N_-1_ corresponds to the residue immediately 5′ of the scissile phosphate (Fig. 1). We refer to recent reviews for discussions of archaeal and eukaryotic RNase P (38, 39, 40, 41, 42).

### RNase P and its substrates: Recognition and versatility

To understand how RNase P recognizes and interacts with pre-tRNA, Altman took advantage of available tRNA^Tyr^Su3 mutants (Fig. 1) coded by bacteriophage Φ80. These were used at the LMB to study and understand tRNA structure and function (7). Early on, it was realized that changes either in the tRNA body or in the 5′ leader influenced the level of mature tRNA *in vivo*. For example, changing residue G15 to A in the tRNA^Tyr^Su3 precursor (pSu3A15) alters the structure such that pSu3A15 accumulates *in vivo*, and it is processed less efficiently by RNase P *in vitro* [Fig. 1*A* (7, 9, 43, 44, 45, 46)]. Moreover, disrupting a base-paired stem structure in pre-tRNAs resulted in less efficient RNase P cleavage *in vitro*. Introducing second-site mutations that restored the base-pairing (or the tertiary structure) improved RNase P cleavage and increased the yield of matured tRNA *in vivo*. McClain *et al*. subsequently identified pre-tRNAs transcribed from the bacteriophage T4 genome [see above (14, 47)], which carries eight tRNA genes, and six of these were identified as dimeric precursor-tRNAs (48, 49). For some of these precursor variants, disruption of the tRNA structure influenced the amount of tRNA produced relative to the WT tRNA as did the absence of the 3′ CCA (16, 50, 51, 52). Together, these studies suggested that RNase P recognizes the tRNA domain of a pre-tRNA (Fig. 1, *A* and *B*). This conclusion was later corroborated *in vitro* by several research groups using different approaches such as genetics, chemical footprinting, and nucleotide analog interference mapping (NAIM) and ultimately when the RNase P-tRNA crystal structure was obtained (33, 53, 54, 55); see below.

RNase P cleavage results in tRNAs having seven bp in the acceptor stems; however, tRNA^His^ and tRNA^SeCys^ are exceptions (56, 57, 58, 59) as they have 8 bp acceptor-stems. In eukaryotes, RNase P cleavage of the tRNA^His^ precursor results in a seven bp-long acceptor-stem, and a guanylyltransferase adds the extra G at the 5′ end to form 8 bp with a single-stranded 3′-CCA end (60). Together, these data emphasized that the tRNA acceptor-stem is an important determinant for RNase P recognition and cleavage.

The importance of the acceptor-stem for RNase P catalysis was further corroborated using chimeric pre-tRNAs where the tRNA^His^ acceptor-stem was introduced into pre-tRNA^Tyr^Su3 and yeast pre-tRNA^Ser^ (61, 62). Experiments by Green and Vold (63) also emphasized the importance of the tRNA acceptor-stem in the processing of a multimeric tRNA precursor (carrying six complete and one incomplete tRNA sequence) *in vitro* by *Bacillus subtilis* (*Bsu*) RPR without the protein component. In collaboration with the Altman laboratory, McClain showed that *Eco* RPR, both with and without the *Eco* RPP, cleaved synthetic model hairpin-loop substrates efficiently at the canonical cleavage site [Fig. 1*C* (64)]. These model substrates represented the tRNA acceptor-stem, T-stem, and the T-loop that forms a well-defined domain in the crystal structure of tRNA (64, 65, 66). This finding is consistent with the importance of the tRNA acceptor-stem in cleavage by RNase P and led to the development of the external guide sequence technology in the Altman laboratory (discussed further below).

**Figure 2:**
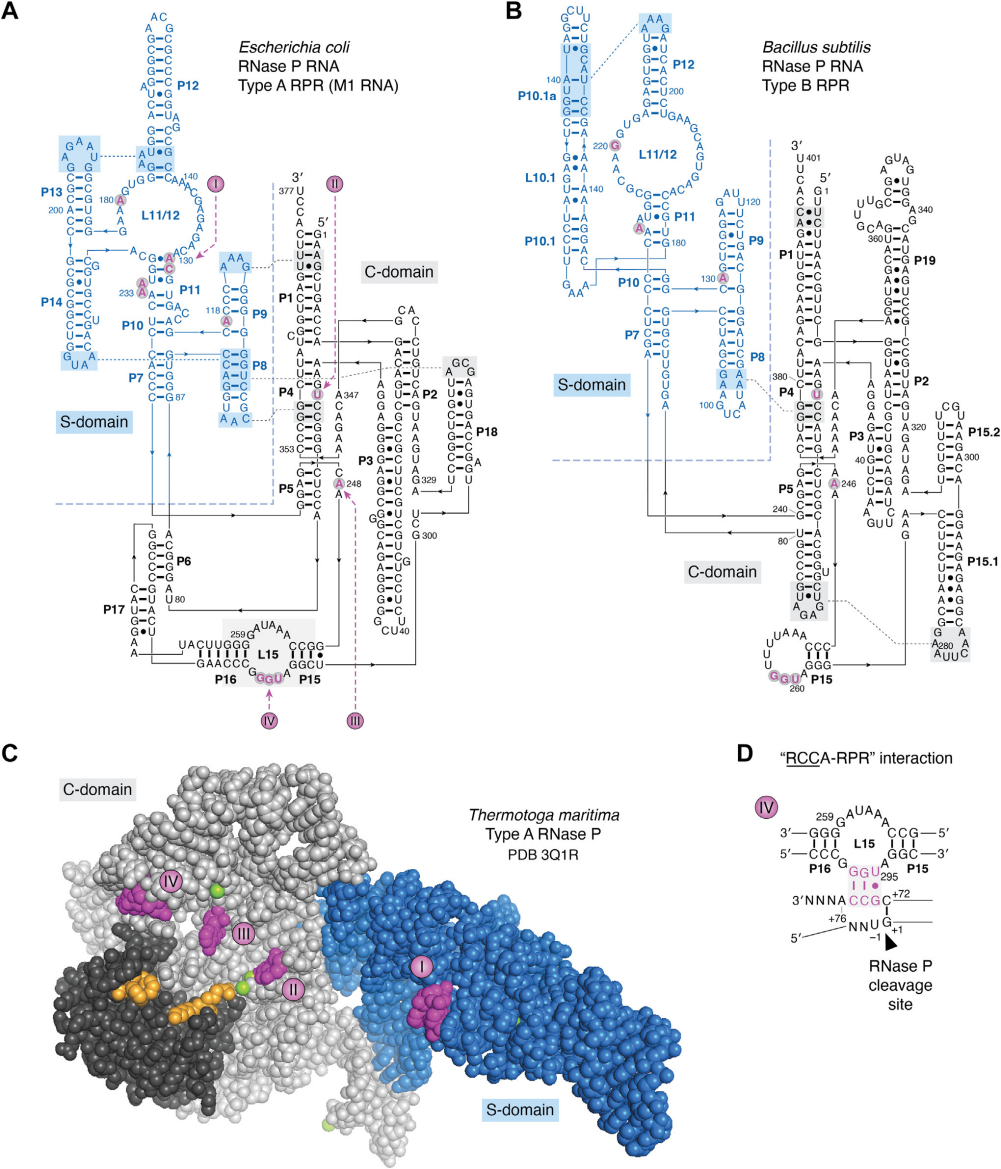
**Structures of the bacterial RNase P RNA and RRP.***A*, the predicted secondary structure of the type A *Eco* RPR (271, 272). The border separating the S- and C-domains is marked with the *dotted line*. Residues highlighted in *magenta* correspond to functionally important residues discussed in the main text, see also Fig. 2*B*: I, residues C_128_, A_129_, A_232_ and A_233_, which are part TSB (the T-loop-stem binding site); II, residue U_69_, which interact with the tRNA acceptor-stem (the "U_69_-amino acid stem interaction") and is involved in coordinating Mg^2+^; III, residue A_248_, which stacks on top of the tRNA G_+1_/C_+72_ pair and is positioned near N_-1_ in the substrate forming the "A_248_/N_-1_" interaction; and IV, residues G_292_, G_293_, and U_294_ which pairs with D_+73_C_+74_ and C_+75_ in the substrate forming the "RCCA-RPR" interaction (highlighted in panel C), D refers to the tRNA discriminator base. The residue C_92_ marked in *orange* is positioned close to C_-3_ in pre-tRNA^Tyr^Su3 as determined by UV-crosslinking (99). For references, see the main text. *B*, the predicted secondary structure of the type B *Bsu* RPR (271, 272). The border separating the S- and C-domains is marked with the *dotted line*. Residues highlighted in *magenta* correspond to residues U_51_, A_130_, G_220_, A_230_, and G_258_-U_260_ [part of the "RCCA-RPR" interaction in type B RPR (116)] discussed in the main text (see above). *C*, the crystal structure of type A RNase P, RPP, from *Thermotoga maritima* (RPR C-domain, *light gray*; RPR S-domain, *blue*; RPP, *dark gray*). Residues marked in *magenta* are mapped on *Eco* RPR in panel A as indicted (spheres in *magenta*, marked I–IV, correspond to the spheres in Fig. 2*A*). The RPP amino acids marked in *orange* interact with a pre-tRNA 5′ leader that was soaked into the crystal. The *green spheres* correspond to metal(II)-ions located near the tRNA G_+1_/C_+72_ and U_69_ in the RPR. The image was created using PyMOL (Schrödinger, LLC) and PDB 3Q1R, although the tRNA was omitted for clarity (33). *D*, illustration of the "RCCA-RPR" interaction (see also Fig. 3*C*). Interacting residues marked in *magenta* and the *arrow* marks the RNase P cleavage site.

Following an *in vitro* evolution protocol, substrates were selected that were processed by *Eco* RPR, with and without *Eco* RPP. Processing of these substrates by *Eco* RPR in the absence and presence of *Eco* RPP agreed with earlier work that the acceptor-stem plays an essential role in the *Eco* RNase P–catalyzed reaction (67). Furthermore, *in vitro* evolution using *Bsu* RPR identified two substrate classes (I and II). Class I carries a stem-loop mimicking the tRNA T-stem/loop (TSL) motif, a single-stranded region in place of the acceptor-stem and a 3′ CCA motif. The class II members have seven base-pairs-long helices appended to either 3′ trailers or to a loop structure. Both these substrate types were cleaved by *Bsu* RPR 5′ of a G residue, in the single stranded region (class I) and in the stem structure (class II). Relative to cleavage of a pre-tRNA^Phe^ substrate, *Bsu* RPR cleaved these variants with less efficiency. Comparing cleavage by *Bsu* RPR and *Eco* RPR showed that cleavage efficiency was lower using *Eco* RPR. The difference was attributed to structural differences between *Eco* RPR and *Bsu* RPR [Fig. 2, *A* and *B*; for details see (68)]. Later, it was demonstrated that both *Eco*- and *Bsu*-reconstituted holoenzymes (RPR assembled with the RPP) cleave short ss RNAs (≥5 residues long) albeit with several orders of magnitudes of lower efficiencies relative to cleavage of pre-tRNAs. As for the *in vitro*–selected substrates, the ssRNAs were cleaved 5′ of guanosines [Fig. 1*C* (69, 70)]. These data are consistent with *Eco* RPR cleaving 5′ of guanosines in single-stranded regions of pre-tRNAs having shortened acceptor-stems with and without RPP (71); these results led to the suggestion that a guanosine at the cleavage site functions as a guiding nucleotide (71, 72). Cleavage of ssRNAs and *Bsu* RPR having 5′ and 3′ extensions by *Bsu* RNase P led to the suggestion that in *B. subtilis* cells, the RNase P is involved in autolytic processing of the RPR transcript (69). In *E. coli*, the processing of the 3′ end of the RPR precursor involves the endoribonuclease E (73, 74).

Together with the finding that RNase P also processes other RNAs (see below), these data uncovered the versatility of RNase P function with respect to substrate recognition and processing. Notably, the demonstration that *Eco* RPR can cleave pre-tRNAs at alternative sites, in particular without the RPP, paved the way to identify key residues in RPR (and in its substrate) playing a role in recognition of the correct cleavage site and to identify the function of the RPP. Moreover, it was clear that *Eco* RPR—with and without the RPP—cleaves model hairpin-loop substrates (64). This result, together with advances in RNA chemical synthesis (75), established the basis for the design and study of cleavage of substrates carrying unnatural nucleobases at selected positions. Several research groups have used this strategy [*e.g*., (76)].

### Bacterial RPR: The S-domain and induced fit

On the basis of the secondary structures, bacterial RPR can be divided into three different types: A (Ancestral type; *e.g*., *E. coli* RPR), B (Bacillus type), and C [*e.g*., *Thermomicrobium roseum*, member of the phylum *Chloroflexi* (41)]. Here, we focus on the functions of the different regions and domains in bacterial type A and type B RPR (Fig. 2, *A* and *B*).

Deletion of *Eco* RPR regions resulted in either loss of activity or catalytically active fragments but with altered substrate specificities. Mixing certain inactive RPR fragments (with nonoverlapping deletions) produced active complexes. Interestingly, deleting residues 94 to 204 of the *Eco* RPR S-domain (Fig. 2*A*) abolishes cleavage of pre-tRNA^Tyr^Su3 (with and without *Eco* RPP), albeit this variant (Δ94–204) still cleaves *Eco* pre-4.5S RNA (a natural RNase P substrate, see below) in the presence of *Eco* RPP (77, 78). Subsequently, Pan suggested that *Bsu* RPR can be divided into two structural domains; the specificity (S-) and catalytic (C-) domains. While neither of these two domains were catalytically active alone, activity was restored when they were mixed together [Fig. 2, *A* and *B* (79)]. Collectively, the data show the importance of the RPR domains irrespective of RPR type. The data also suggest that binding and cleavage of pre-4.5S RNA, unlike pre-tRNA, do not require an intact S-domain for processing by *Eco* RNase P (77, 78). However, it was subsequently demonstrated that bacterial types A and B RPR C-domains lacking the S-domain are catalytically active, with and without the RPP, even with pre-tRNAs as substrates although pre-tRNA binds the C-domain with less affinity (80, 81, 82, 83, 84).

**Figure 3:**
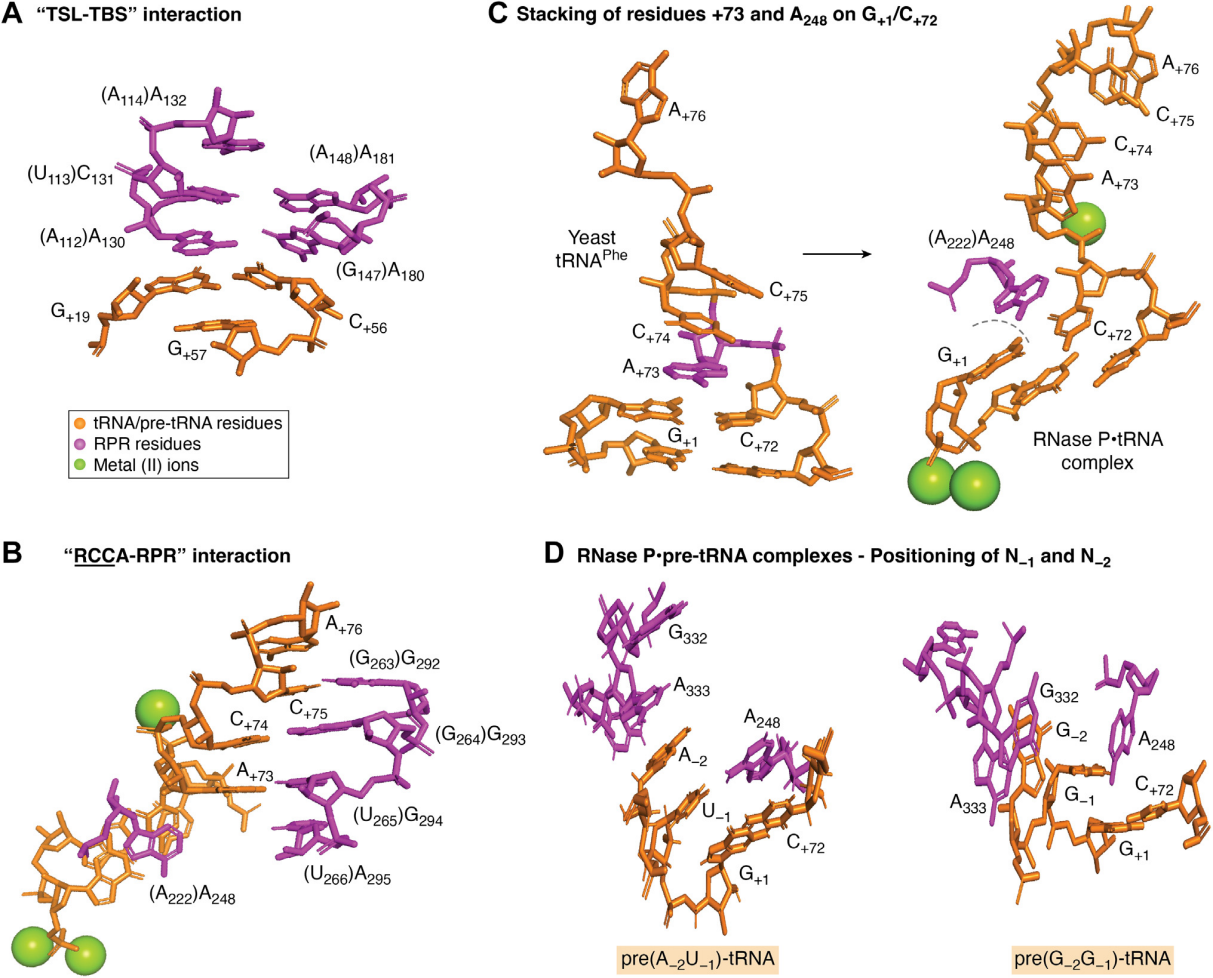
**Stacking interactions and base pairing between bacterial type A RPR and tRNA/pre-tRNA.** tRNA/pre-tRNA residues are colored in *orange* and RPR residues in *magenta*. The numbering corresponds to *Eco* RPR (see Fig. 2*A*) while residues in parenthesis refers to the *Thermotoga maritima* RPR numbering in the RNase P-tRNA crystal structure. *A*, stacking between the tRNA TSL-region and residues in the TBS in the S-domain as observed in the RNase P-tRNA structure. *B*, the "RCCA-RPR" interaction in the RNase P-tRNA structure, see also Fig. 2*D*. The *green spheres* represent metal(II) ions. *C*, *left* panel, stacking of the discriminator base (A_+73_, colored in *magenta*) on the yeast tRNA^Phe^ G_+1_/C_+72_. *Right* panel, stacking of A_248_ (*Eco* RPR numbering) on the tRNA G_+1_/C_+72_ pair in the RNase P-tRNA structure. The *green spheres* represent metal(II) ions. *D*, interactions between N_-1_/N_-2_ in the pre-tRNA 5′ leader and *Eco* RPR in the cryo-EM RNase P-pre(A_-2_U_-1_)-tRNA (*left* panel) and RNase P-pre(G_-2_G_-1_)-tRNA (*right* panel) structures. For *Eco* RPR residues numbering, see. 2A. The images were created using PyMOL (Schrödinger, LLC), PDB 1EHZ [yeast tRNA^Phe^ (273)], PDB 3Q1R (33), PDB 7UO1 [cryo-EM, RNase P-pre(A_-2_U_-1_)-tRNA (37)], and PDB 7UO0 [cryo-EM, RNase P-pre(A_-2_U_-1_)-tRNA (37)]. TSL, T-stem loop.

Before the RNase P-tRNA crystal structure was resolved, cross-linking, modification interference, and genetic and biochemical data suggested that particular residues and chemical groups in the pre-tRNA TSL region interact with the RPR S-domain (both type A and B) (82, 85, 86, 87, 88, 89, 90, 91, 92). Specifically, it was suggested that 2′-OH groups in the T-loop/stem at positions 54, 56, 61, and 62 (see Fig. 1, *A* and *B*) influence binding of pre-tRNA to the type B *Bsu* RPR (91, 93). In addition, the exocyclic amine at position 4 of C_56_ was postulated to interact with the RPR. These chemical groups were suggested to interact with residues in the P11 region in the S-domain (Fig. 2). In particular, the data indicated that the 2′-OH at position 62 interacts with A_230_ of *Bsu* RPR (corresponding to A_233_ in *Eco* RPR; Fig. 2, *A* and *B*). Other RPR residues in the S-domain suggested to contact the TSL region were A_130_ and G_220_ (in *Bsu* RPR), corresponding to A_118_ and A_180_ in *Eco* RPR [Fig. 2, *A* and *B* (91, 93, 94)]. The bacterial RNase P-tRNA crystal structure (33) later revealed that base stacking is a main contributor to the interaction between TSL (the "tRNA elbow") and the RPR. The RPR "base stacking" platform motif is formed by two intertwined T-loops (95), which include residues A_130_/C_131_ and A_180_/A_181_ [*Eco* RPR numbering; Figures 2*A* and 3*A* ((95), see also (96))]. The TSL-binding site is referred to as TBS, and the interaction is referred as the TSL–TBS interaction.

Assays using pre-tRNA^Phe^ variants with 2′-OH changed to 2′-H substitutions at specific positions in the TSL region and at the cleavage site revealed reduced cleavage by *Bsu* RPR compared to the unmodified substrate (91). It was inferred that catalysis is dictated also by interactions distal to the cleavage site. A conformational change/spatial rearrangement upon *Bsu* RPR–pre-tRNA complex formation was postulated to depend on the TSL–TBS interaction (82). Furthermore, *Eco* RPR cleaves model hairpin-loop precursors based on the acceptor-stem, T-stem, and T-loop of the *E. coli* pre-tRNA^Ser^Su1; this cleavage occurs preferentially at the correct site with and without the *Eco* RPP [see *e.g*. (97)]. Replacing the "T-loop" with tetra-loop variants reduces cleavage efficiency and shifts the cleavage site such that cleavage by *Eco* RPR occurred between N_-2_ and N_-1_ in the 5′ leader: correct cleavage occurs 3′ of N_-1_, between N_-1_ and N_+1_ (Fig. 1). Interfering with the TBS structural topology (by mutating G_125_ and/or C_235_ in the P11-region; Fig. 2*A*) restored cleavage efficiency at the correct site leading to the suggestion that efficient and correct cleavage depends on a productive TSL–TBS interaction. These and other data provided evidence for an induced-fit mechanism for bacterial RPR-mediated cleavage [(98) see also (99)]. We also note that NAIM data suggest overlapping but not identical binding modes for pre-tRNA and mature tRNA using *Eco* RPR. Strong interference was detected at positions G_19_, G_53_, A_58_, and G_71_ [Fig. 1 ((100), see also (101))]. Notably, Bothwell *et al.* (102) postulated that RNase P interacts with T-loop of the pre-tRNA and uses a measuring device to identify the cleavage site [see also (89, 103, 104)]. The "Bothwell postulate" (102) also agrees with the discussion above and that the distance between the T-loop and the tRNA 5′ terminus corresponds to the conserved 12 bp in tRNAs (Fig. 1, *A* and *B*).

In keeping with the induced-fit theory (105), a productive TSL–TBS interaction (see above) is suggested to induce a conformational change in the RPR–substrate complex, in particular at the cleavage site thereby affecting site selection and cleavage rate (98). Details of this signaling at the structural level are at present not known; however, analysis of interactions that connect the S- and C-domains might be informative (33, 106, 107, 108). Neither interfering with the P8/P18 contact, which is important for the connection between the S- and C-domains [Fig. 2*A* (33, 109, 110)], nor deleting P18 (ΔP18-variant) changed the cleavage site as assessed using a model substrate; however, the cleavage efficiency was significantly reduced in these mutants (84). The cleavage rate (k_obs_) for the ΔP18-variant was >3000-fold lower relative to WT *Eco* RPR; the K_D_ for substrate binding was unchanged [see also (106, 107, 108)]. Also, the data obtained using a substrate carrying a 2′-NH_2_ at N_-1_ suggested that interference with the P8–P18 interaction, the structure near the TBS, or deleting the S-domain influenced the charge distribution at the cleavage site (84). Protonation of the 2′-NH_2_ (at N_-1_) at lower pH produces a positive charge at the cleavage site resulting in a lower cleavage frequency at the correct site. At higher pH, cleavage at the correct site increases, reflecting the deprotonation of this 2′-NH_3_^+^. The positively charged 2′-NH_3_^+^ would interfere with Mg^2+^ binding at and in the vicinity of the cleavage site [see below and (111, 112)]. Together, these data suggest a role for the P8–P18(L18) interaction and P18 with respect to a productive TSL–TBS interaction (Fig. 2*A*) and the structural architecture at (and near) the cleavage site that ensures correct and efficient cleavage.

### Bacterial RPR and the "RCCA–RPR" interaction

Many tRNA genes in bacteria contain the universally conserved 3′ CCA motif (113). Early studies indicated that the 3′ CCA motif of tRNA precursors affected RNase P cleavage [see above (16, 43, 50, 51)]. By studying the choice of cleavage site, it was later revealed that the 5′ GGU motif in the P15 loop in *Eco* RPR (Fig. 2) base pairs with the 3′ "RCCA-motif" [interacting residues underlined and R corresponds to the tRNA discriminator base at position 73 (114), Fig. 1, *A*–*C*]. This pairing is referred to as the "RCCA–RPR" interaction. It is present both with and without *Eco* RPP (115, 116) and is supported by footprinting as well as single-turnover kinetic data (94, 117, 118). The interaction is also observed in type B RPR substrate complexes (116, 119), and it is essential for catalysis in *E. coli* and *B. subtilis* cells (119, 120). The RNase P-tRNA crystal structure confirmed the "RCCA–RPR" interaction (33).

The "RCCA–RPR" interaction was suggested to anchor the substrate to the RPR, expose the cleavage site, and result in re-coordination of Mg^2+^ at and in the vicinity of the cleavage site to ensure accurate and efficient cleavage [(115); see also below]. This idea is consistent with the finding that small RNAs representing the *Eco* RPR P15 loop (Fig. 2*A*) and P15-P15.1 of type B RPR (Fig. 2*B*) mediate cleavage of both pre-tRNA and a model hairpin loop substrate each at the correct site, but with reduced efficiency (121). This observation, together with the finding that the group II intron domain V (as part of small RNAs) catalyzes hydrolysis of the exon-intron junction in trans (122), provided evidence that complex RNAs like the RPR and rRNAs are composed of functional domains (123).

### Bacterial RNase P: Importance of the RPP/C5 protein and the 5′ leader

The number of protein subunits varies in RNase P: one in bacteria (RPP), four to five in archaea, and nine to ten in eukaryotes (53). As discussed above, the isolation of temperature-sensitive (*ts*) *E. coli* strains carrying mutations in the C5/RPP protein gene, *rnpA*, suggested that the *Eco* RPP is essential for activity *in vivo* (18, 19, 124, 125). Sequencing later confirmed that these *E. coli* strains indeed carry changes in *rnpA*, C5^A49^ (R46H), and C5^ts241^ (E71K) (45). Several overexpression protocols were tested to generate significant amounts of the *E. coli* C5 (and C5^A49^) protein; the Altman laboratory developed protocols using the T7 RNA polymerase–based protein overexpression system [(126, 127); see also (128)]. This work led to the demonstration of the 1:1 stoichiometry (of the RPP and RPR) in the RNase P holoenzyme, determination of the dissociation constant for the interaction between *Eco* RPR and *Eco* RPP, and mapping of regions in *Eco* RPR that interact with *Eco* RPP (78, 126, 129). Reconstitution experiments further suggested that the *ts*-phenotype associated with C5^A49^ is caused by a defect in the assembly of the RNase P holoenzyme (130). This conclusion agrees with the observation in several laboratories that the *E. coli* A49 *ts*-phenotype can be rescued by increasing the levels of RPR *in vivo*. Later findings showed that overexpressing other *Eco* RPP mutant proteins also rescues the A49 *ts*-phenotype and provided models of RPR in complex with RPP. Interestingly, single amino acid substitutions in the RPP alter substrate specificity of the reconstituted RNase P holoenzyme (131). Experiments using RPR with deletions or single nucleotide mutations indicated that the RPR tertiary structure is a determinant for RPP recognition. Moreover, based on footprinting techniques, RPP-RNA contact sites in *Eco* RNase P were identified, and these data were used to generate structural models of the bacterial RNase P holoenzyme (132, 133, 134). Understanding RPR–RPP interactions in RNase P (type A) became possible with the availability of the crystal structure of the RNase P-tRNA complex (Fig. 2*C*). The RNase P-tRNA crystal structure and models of the complex are in good agreement (33, 133, 134).

The RPP lowers the Mg^2+^ requirement and increases cleavage efficiency *in vitro*, and it was suggested that the RPP acts as an electrostatic shield (23, 59). Subsequent studies showed that the RPP stabilizes the catalytic active RPR conformation (135, 136), modulates substrate specificity (53, 137, 138, 139), and affects the metabolic stability of the RPR (140). Binding of the RPP to the RPR also affects RPP solubility and proteostasis in the cell (141, 142).

The structure of the RPP revealed a cleft and FRET as well as other biochemical data suggested that the RPP interacts with N_-8_ - N_-3_ in the "single-stranded" 5′ leaders and influence the positioning of the 5' leader in the RNase P-substrate complex [see also above and Fig. 1*A* (143, 144, 145, 146, 147, 148)]. Binding the 5′ leader stabilizes the RNase P-substrate complex (143, 149). The length of the 5′ leader, however, varies with pre-tRNA identity; moreover, the length affects binding and the association rate constant, but not the cleavage rate constant [(138, 149), but see (150)]. These observations are consistent with *in vivo* data suggesting that the 5′ leader structure or length correlates with bacterial growth rate [(151), see also Chamberlain *et al.*, this issue]. Furthermore, the interaction of the RPP with the 5′ leader affects the affinity for metal ions necessary for cleavage. The interaction also compensates for structural differences among tRNA precursors by altering the energetic contributions to 5′ leader binding (138, 147, 152, 153).

Recent High Throughput Sequencing-Kinetics, HTS-Kin, data indicate that the *Eco* RPP facilitates the recognition of a consensus sequence in the pre-tRNA 5′ leaders [(154, 155, 156) and Chamberlain *et al.*, this issue]. Briefly, the identity of the N_-3_ and N_-2_ in the 5′ leader influences the selection of alternative substrates at the association step and not the cleavage step; also, the N_-2_ identity can influence the binding contribution of the RPP [(156, 157); see also below]. In addition, a sequence-favored interaction was reported between N_-4_ in the 5′ leader and the RNase P protein (158, 159). This finding may explain how substitution of a C to U at −4 in the 5′ leader of pre-tRNA^Tyr^Su3 rescues the lower suppression efficiency caused by the G_+2_ to A_+2_ substitution (see below; Fig. 1*A*). Collectively, these data emphasized the importance of the pre-tRNA 5′ leader for correct and efficient RNase P processing and the contribution of RPP to the cleavage reaction.

### Bacterial RPR: The "A_248_–N_-1_" interaction and residue N_-2_

Examination of the bacterial tRNA gene sequences did not reveal any conserved sequences in 5′ leaders that could potentially interact with the RPR [see *e.g*. (154), and Chamberlain *et al.*, this issue]. However, in many bacteria, U_-1_ in pre-tRNA 5′ leaders (Fig. 1*A*) is the most abundant residue; in *E. coli*, roughly 60% have U_-1_. The adenosine at position 248 (A_248_; *E. coli* numbering; Fig. 2*A*) is conserved among bacterial RPRs and it has been suggested that A_248_ forms a Watson-Crick (WC) base pair with residue U_-1_ in the pre-tRNA leader (160, 161). Several *E. coli* precursors do not have U_-1_. Also, in certain high GC-content bacteria, such as mycobacteria, C_-1_ is more frequent than U_-1_, whereas in some other bacteria, G_-1_ and A_-1_ are also more abundant than U_-1_ (162). Moreover, WT *Eco* RPR cleave model hairpin-loop substrates having 3-methyl-U_-1_, which interferes with the U_-1_ WC-surface, at the correct site [(163, 164); for a detailed analysis of the potential N_-1_/N_248_ WC base pairing, see (164)]. Together, these data argue against WC-base pairing between N_-1_ (pre-tRNA) and A_248_ (RPR). In this context, the presence of a nucleobase at −1 is not required as suggested from studies of a model substrate but its absence results in aberrant cleavage and a reduction in the cleavage efficiency (76).

The bacterial RNase P-tRNA crystal structure represents the post-cleavage state and therefore provides no information about the interaction between N_-1_ and A_248_ in the RNase P-substrate ground-state complex (33). However, the Harris laboratory recently reported cryo-EM structures for *Eco* RNase P in complex with pre-tRNAs having U_-1_ (and A_-2_) or G_-1_ (and G_-2_) (37). The crystal and cryo-EM structures show that A_248_ stacks on the tRNA G_+1_/C_+72_ bp (Fig. 3, *C* and *D*). This stacking interaction depends on the N_-1_ identity: for pre-tRNA with U_-1_ (and A_-2_), A_248_ stacks on G_+1_/C_+72_, while for substrates with G_-1_ (and G_-2_), A_248_ is positioned orthogonal relative to G_+1_/G_+72_ (Fig. 3*D*). For the pre(A_-2_U_-1_)-tRNA, A_-2_, A_333_, and G_332_ forms a continuous stack on U_-1_ (Fig. 3*D*). In the case of pre(G_-2_G_-1_)-tRNA, G_-1_ appears to stack on the pre-tRNA G_+1_ with A_333_ and G_332_ stacking on G_-2_ (Fig. 3*D*). Moreover, the *Eco* RNase-pre-tRNA cryo-EM structures do not show WC pairing between N_-1_ and A_248_.

In the bacterial RNase P-tRNA(-pre-tRNA) complexes, the Hoogsteen surface of A_248_ faces the 5′ termini of the tRNA [Fig 3, *C* and *D* (33, 37)]. Cross-linking and NAIM data indicate that the region at and near A_248_ interacts with the RPR and that the A_248_ Hoogsteen surface contributes to substrate binding (53, 165, 166, 167, 168, 169). This inference raises the possibility that N7 and the 6-NH_2_ of A_248_ interact with chemical groups of N_-1_. Consistent with this notion, the *Eco* RNase P-pre(A_-2_U_-1_)-tRNA cryo-EM structure (37) suggests that the O4 of U_-1_ is positioned to form H-bonds with the A_248_ exocyclic amine. With G_-1_, hydrogen bonding might be formed between O6 of G_-1_ and 6-NH_2_ of A_248_ (Fig. 3*D*; see also Chamberlain *et al.*, this issue).

Changing G_+2_ to A_+2_ in the pre-tRNA^Tyr^Su3 acceptor-stem reduced the level of matured tRNA *in vivo*, while a "second-site" mutation in the 5′ leader, C_-4_ to U_-4_ increased the tRNA levels five- to six-fold [Fig. 1*A* (8, 10, 20)]. Mutating G_-2_ and U_-1_ in pre-tRNA^Tyr^Su3 to C_-2_ and A_-1_ did not change the cleavage site selection *in vivo* or *in vitro*. However, the resulting tRNA^Tyr^Su3 suppressed UAG with ≈seven-fold lower suppression efficiency than the "WT" tRNA^Tyr^Su3 (170). Together, these findings indicate that structural changes in the pre-tRNA^Tyr^Su3 5′ leader can influence processing *in vivo* (see also Chamberlain *et al.*, this issue). Furthermore, *in vitro* studies revealed that the cleavage site varies depending on the identity of N_-2_ in the 5′ leader of pre-tRNAs and RPR type [type A *Eco* RPR and type B *Mycoplasma hyopneumoniae* RPR, *Hyo* RPR (171)]. Cleavage kinetics was also affected by the identity of the −2 residue in the substrate and with RPR type. Pre-tRNA with G_-2_ was cleaved with the lowest rate with the type B *Hyo* RPR, while the type A *Eco* RPR cleaved the U_-2_ pre-tRNA with the lowest rate (172). Hence, comparing *Eco* RPR (type A) and *Hyo* RPR (type B) reveals differences both with respect to cleavage site selection and cleavage rate (see also above). Also, for *Eco* RNase P, the G_-2_ in pre-tRNA^fMet^ affects site selection and compensates for the negative impact by its WT C_+1_/A_+72_ wobble bp (173, 174). Together, these *in vivo* and *in vitro* data identify N_-2_ as important for correct and efficient RNase P cleavage [see also (171)]. In this context, an *R*p-phosphorothioate modification at −2 in a tRNA^Gly^ precursor interfered with cleavage by the *Bsu* RNase P holoenzyme indicating that it interacts, either indirectly (*via* a metal(II) ion) or directly with the enzyme (76). As discussed below, Mg^2+^ binds near the cleavage site (69, 175, 176). It is therefore conceivable that the N_-2_ nucleobase can influence the positioning/binding of Mg^2+^ and thereby impact substrate binding and/or catalysis (see also below).

Together, these data suggest that the stacking interactions at and near the cleavage site play important roles in anchoring the substrate where A_248_, G_332_, and A_333_ are part of a binding surface for N_-2_ and N_-1_ [(37), see also (53)]. The structural data further suggest that the architecture of this binding surface depends on the identities of N_-2_ and N_-1_ (Fig. 3*D*).

### Chemical groups near the cleavage site and role of Mg^2+^

Understanding the functions of different RNAs is tightly linked to deciphering the roles of metal ions such as Mg^2+^; on average, there is one Mg^2+^ bound per 3 to 4 nucleotides [see *e.g*., (177, 178, 179)]. Binding of metal ions affects RNA folding, RNA–RNA interactions, RNA–protein interactions, and catalysis. Their role in RNA folding has been covered in previous reviews (180, 181) and will not be discussed here. Here, we emphasize that bacterial RPR is folded into an active structure in a cooperative process that is completed at 5 to 10 mM Mg^2+^ [for reviews, see *e.g*., (180, 181)].

As discussed above, RNase P activity with and without the protein subunit depends on the presence of metal(II) ions, that is, Mg^2+^. Mg^2+^ affects the folding of the RPR and is involved in the chemistry of the cleavage reaction. For activity, however, Mg^2+^ can be replaced by other metal(II) ions such as Mn^2+^ and Ca^2+^ (177). Cleavage by RPR without the protein requires a higher concentration of metal(II) ions than when the RPP is present [see above (30, 182, 183); notably, addition of spermidine lowers the Mg^2+^ requirement in the RPR alone reactions (183)]. Under certain conditions, binding of metal(II) ions to an RNA cleaves the phosphodiester back bone by activating a neighboring 2′-OH as a nucleophile generating 5′-OH and 2′,3′-cyclic phosphate as cleavage products (184). Kazakov and Altman (175) showed that *Eco* RPR is cleaved by Mg^2+^ at five specific positions at pH 9.5, suggesting that these cleavage sites are in close proximity to where Mg^2+^ binds. Others have used Pb^2+^ to map metal(II) ion binding sites in both type A and type B RPRs and to probe the integrity of the RPR structure in response to changing residues in the RPR (185, 186, 187). Together, these studies placed Mg^2+^-binding sites in the vicinity of where the tRNA TSL region interacts in the S-domain and where the pre-tRNA 3′ RCC-motif interacts with *Eco* RPR (P15 loop; Fig. 2*A*). Metal(II) ions were subsequently detected at these and other sites and at sites in the proximity of the tRNA 5′ end in the RNase P-tRNA crystal structure and the *Eco* RNase P-pre-tRNA cryo-EM structures [Figs. 2 and 3 (33, 37)].

Under certain conditions, Mg^2+^ also induces cleavage of pre-tRNA^Tyr^Su3 between C_-3_ and G_-2_ in the 5′ leader, that is, at the junction of single- and double-stranded regions [Fig. 1*A* (175)]. Furthermore, based on the observed Mg^2+^-induced cleavage of model substrates, in which the 2′-OH had been replaced by 2′-H at U_-2_, C_-1_, G_+1_, and C_+33_ (C_+33_ in the AT1 model substrate corresponds to C_+74_ in pre-tRNA; Fig. 1*C*), it was suggested that the true substrate for *Eco* RPR has Mg^2+^ coordinated at the junction between the single- and double-stranded regions [Fig. 1*C* (72, 176)]. The structural topography of N_+1_/N_+72_ also appears to influence binding metal(II)-ion(s) in the vicinity of the cleavage site (188, 189).

The 2′-OH at the cleavage site (at N_-1_) is not required (190) but it influences different reaction steps. Substituting the 2′-OH at N_-1_ with 2′-H, 2′-F, or 2′-NH_2_ in various RNA substrates, several laboratories have demonstrated that it (and its binding to Mg^2+^) has a role in ground state binding of the substrate, cleavage site recognition/selection, and cleavage efficiency (55, 177). For example, *Eco* RPR-mediated cleavage of yeast tRNA^Phe^ extended with a deoxyA_-1_ led to a lower cleavage rate with only a small effect on substrate binding compared to the unmodified substrate [steady state conditions (191)]. Smith and Pace suggested that ≥3 Mg^2+^ are bound near the cleavage site and are required for optimal cleavage (191). Replacing the N_-1_ 2′-OH group with 2′-H decreased the number of Mg^2+^ from three to two, indicating that this 2′-OH may be involved in binding Mg^2+^ during catalysis (see also above). Kazakov and Altman (175) suggested participation of two Mg^2+^ in the cleavage mechanism and two (rather than three) metal(II) ions were detected by cryo-EM and in the crystal structure near the cleavage site (see below).

Studies using *Bsu* RPR and a yeast pre-tRNA^Phe^ with a five-nt-long 5′ leader also indicated the importance of the 2′-OH at position −1 for cleavage, both at the correct and alternative sites. In contrast, the 2′-OH at alternative cleavage sites does not significantly influence catalysis (91, 92). For *Eco* RPR, cleavage of model substrates with 2′-H (at N_-1_) at the correct site does not affect mis-cleavage at the alternative site. This finding is consistent with the idea that the 2′-OH in the immediate vicinity of the cleavage site affects Mg^2+^ binding (163). However, a possible interaction of this 2′-OH with the RPR cannot be excluded. This study also suggested a greater dependence on the 2′-OH at the cleavage site in the absence of the interaction between substrate residues +73 and 294 in the RPR (part of the "RCCA–RPR" interaction, see above). Moreover, it has been suggested that the 2′-OH at the cleavage site acts as an outer (or inner) sphere ligand for Mg^2+^ in the RNase P-substrate complex [(111, 112) see also (161)]. Evidence that the N_-1_ 2′-OH is involved in Mg^2+^ binding at the cleavage site is supported by data using substrates carrying a 2′-NH_2_ at N_-1_. The frequency of cleavage at N_-1_ vs N_+1_ (see Fig. 1) was shown to depend on pH; at pH 5.5, cleavage was detected at −1 while the cleavage site shifted to +1 with increasing pH. As discussed above, the presence of 2′-NH_3_^+^ at the cleavage site would interfere with Mg^2+^ binding (111, 112). The deprotonation of 2′-NH_3_^+^ at N_-1_ is also influenced by the identity of the +1/+72 bp in the substrate, indicating its role in positioning Mg^2+^ at the cleavage site [(189) see also (84, 164)]. Interestingly, in the *Eco* cryo-EM RNase P-pre(A_-2_U_-1_)-tRNA structure, Ca^2+^ appears to be coordinated to the U_-1_ 2′-OH (37). Together, these data suggest that the N_-1_ 2′-OH is involved in binding/positioning Mg^2+^ near the cleavage site.

Two putative Mg^2+^ ions are positioned at the cleavage site in the yeast RNase P-pre-tRNA cryo-EM structure and both are coordinated to the *R*p-oxygen at the cleavage site (35; see also above). In the bacterial RNase P-tRNA crystal structure, two metal ions are also located near the matured 5′-end of the tRNA [Figs. 2 and 3 (33)]. Substitution with sulfur of either of the *R*p- or *S*p-oxygen at the cleavage site in pre-tRNAs led to slower cleavage rates by *Eco* RPR. Replacing Mg^2+^ with the "thiophilic" Cd^2+^ (or Mn^2+^) rescues cleavage of the pre-tRNA with the *R*p-phosphorothioate modification (192, 193, 194). These data suggest a direct role of the *R*p-oxygen in coordinating Mg^2+^ at the cleavage site. In addition, residues in (and near) the P4-helix are close to the two metal ions positioned at the tRNA 5′ termini in the RNase P-tRNA crystal structure. The sequence of the P4-helix is well-conserved; P4 contains a metal(II)-binding site where a bulged U_69_ (Fig. 2) binds the catalytic metal ion. Cross-linking data suggested that U_69_ indeed interacts with the tRNA acceptor-stem; this "U_69_-acceptor stem" interaction has been suggested to influence the affinity of catalytic metal ion(s) at the cleavage site through substrate positioning (195).

### Substrate interaction, base stacking, and mechanism of RNase P cleavage

The "mature" tRNA structure is already adopted in the tRNA precursor (196, 197). As discussed above, the TSL region and the 3′ RCCA-motif interacts with RNase P and the length of the T- and acceptor-stem (12 bp-long in tRNAs) is suggested to act as a measuring device (for references, see above) that helps to determine the RNase P cleavage site. Continued base stacking involving bases both in TSL and TBS (Fig. 3*A*) together with the pairing between the tRNA "3′ RCCA-motif" and GGU sequence in the P15 loop anchors the substrate [Figs 2*D* and 3*C* (33, 164)]. Both A_76_ at the tRNA 3′ end and residue U_257_, which corresponds to A_295_ in *Eco* RPR (Fig. 2*A*), stack on the "RCC-U_294_G_293_G_292_-helix" (Fig. 3*B*). Moreover, the yeast tRNA^Phe^ structure suggests that the discriminator base stacks on top of G_+1_/C_+72_, forming a structural unit (Fig. 3*C*, left panel). Formation of the "RCCA–RPR" interaction results in pairing of the discriminator base at position +73 in the tRNA and U_294_ and stacking of A_248_ on top of G_+1_/C_+72_ in the RNase P-tRNA crystal and *Eco* RNase P-pre(A_-2_U_-1_)-tRNA cryo-EM structures [Fig. 3, *C* and *D* (33, 37)].

These results suggest that base stacking plays an important role in stabilizing the RNase P-substrate interaction. In addition to its involvement in anchoring the substrate, it has been suggested that the stacking of A_248_ on top of the G_+1_/C_+72_ bp acts as a cap. The cap would prevent water accessing the hydrophobic tRNA acceptor-stem and promoting nonspecific hydrolysis. The same expectation holds for the "RCCA–RPR interaction" [Fig. 3*B* (164)]. Moreover, in bacterial RPRs, A_248_ (*E. coli* numbering) is conserved and the stacking free energy for adenosine is more advantageous relative to G, C, and U (198). Recent data also suggest that WT *Eco* RPR_A248_ has the lowest activation energy barrier than *Eco* RPR variants with G, C, or U at position 248 (164). Collectively, these data provide a rationale for the conservation of A_248_ in bacterial (and some archaeal) RPR.

**Figure 4:**
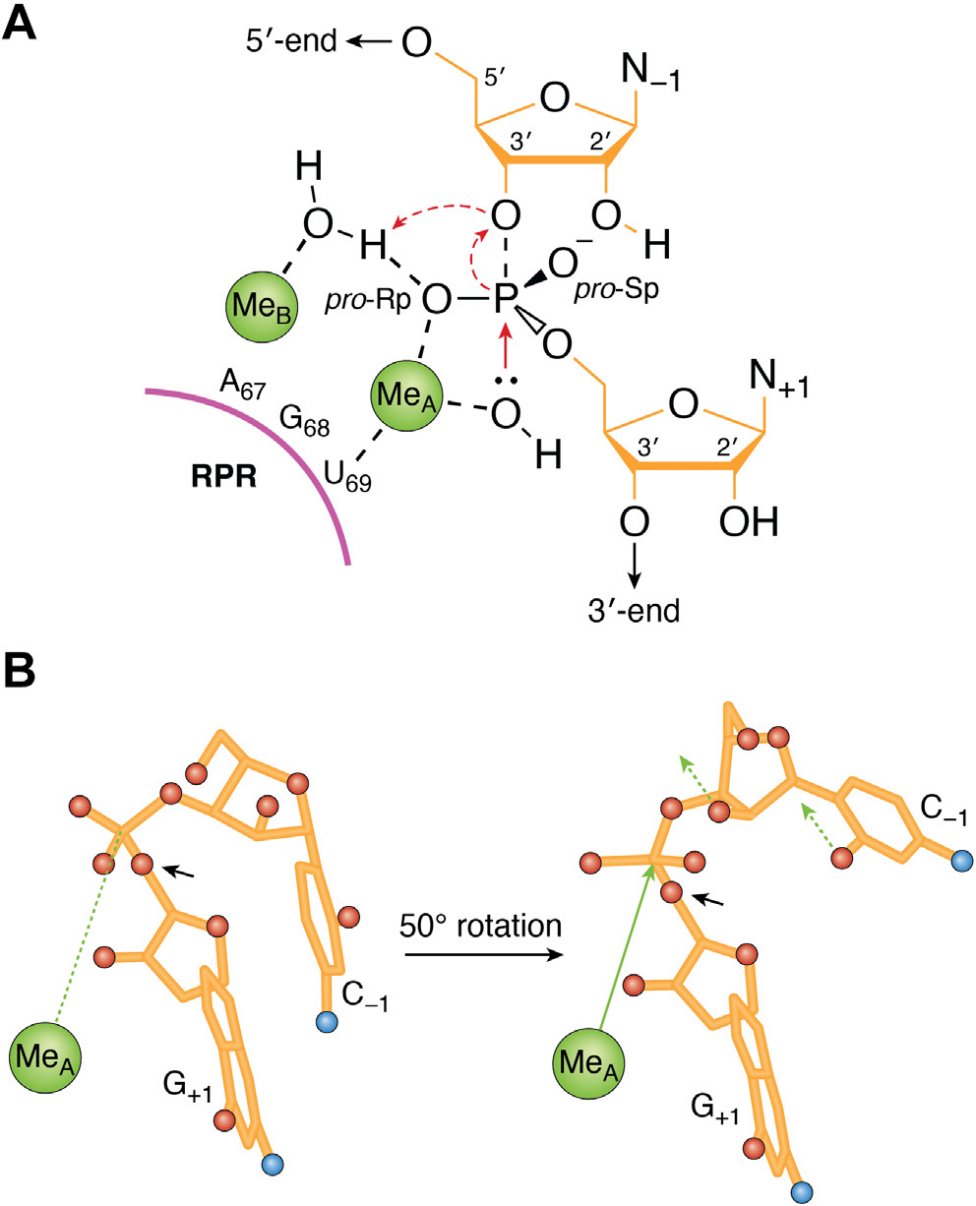
**Models of the RNase P reaction mechanism.***A*, the model is adapted based on the models proposed by Liu *et al.* (201) and Wan *et al.* (36). The substrate (*orange*), the RPR (*magenta*), and metal(II) ions, Me_A_ and Me_B_ (*green*), are color coded as in Figure 1, Figure 2, Figure 3. The RPR numbering refers to *Escherichia coli* RPR, see Fig. 2*A*. The *solid red arrow* marks the nucleophilic attack on the phosphate, while the *dashed red arrows* mark the leaving 3′OH group and its protonation. *B*, model showing a 50^°^ rotation of the P-O5′ phosphodiester bond (*blue arrow*) at the RNase P cleavage site, in a substrate with C_-1_ and G_+1_, that position the Mg^2+^ that activates the water molecule for a nucleophilic attack on the phosphate as indicated (*solid green arrow*). The *green dashed arrows* mark the C_-1_ O2 and the 2′-OH groups, which are suggested to bind Mg^2+^ (see main text). These two groups are pointing in the same direction and both contribute to catalysis (76). The *red*- and *blue*-filled circles mark oxygens and exocyclic amines, respectively. Model adapted from Wu *et al.* (76).

RNase P cleavage generates 3′-hydroxyl and 5′-phosphate products. Two metal(II) ions have been identified in the structures of various RNase P in complex with tRNA [post-cleavage states of bacterial, archaeal, and human RNase P (33, 35, 36) and with pre-tRNA yeast RNase P (34) and *Eco* RNase P (37)]. Irrespective of the RNase P source, two metal(II) ions are positioned near the 5′ end of the tRNA, suggesting a general two-metal-ion catalytic mechanism (Fig. 4*A*). However, before the structures became available, mechanistic models involving Mg^2+^ were presented based on available biochemical data and similarities to cleavage by other ribozymes and protein nucleases [see *e.g*., (159, 175, 183, 191, 192, 193, 199, 200, 201). The combined biochemical and structural data led to the suggestion that the cleavage reaction proceeds through a pentacoordinate transition state. One metal(II) ion activates a water molecule that acts as the nucleophile (Me_A_; Fig. 4*A*). The other metal(II) ion (Me_B_) stabilizes the developing oxyanion in the transition state and might be involved in mediating the transfer of a proton resulting in a 3′-OH on the 5′ leader product. The conserved residue U_69_ (*Eco* RPR numbering; Fig. 2*A*) is implicated as playing a role in positioning the Mg^2+^ that generates the nucleophile, a model that is consistent with earlier biochemical and genetic data [Fig. 4*A*, see above and *e.g*. (159, 200, 201)]. As discussed above, the 2′-OH at N_-1_ in the substrate is also suggested to be involved in Me(II) ion binding (Me_B_; Fig. 4*A*). The carbonyl oxygen (O2) at position 2 on the nucleobase (U_-1_ and C_-1_) contributes to cleavage by *Eco* RPR. Modeling and the *E. coli* cryo-EM RNase P-pre(A_-2_U_-1_)-tRNA complexes suggest that this O2 is exposed on the same surface as the N_-1_ 2′-OH (37, 76). This feature raises the possibility that this oxygen also contributes to binding of Me_B_ (76). In this scenario, however, the N_-1_ 2′-OH has to be prevented from acting as the nucleophile, as it would generate incorrect cleavage products with 5′-OH and 2';3′-cyclic phosphate at their termini [(111, 112) see also (161)]. Modeling suggests that this outcome is achieved by the N_-1_ 2′-OH, pointing away from the scissile phosphate [Fig. 4*B*; for a detailed discussion (76)]. In this context, the N_-1_ 2′-OH is facing in a different orientation relative to the scissile phosphate in the *Eco* RNase P-pre-tRNA cryo-EM structures (37).

### Processing of dimeric and multimeric precursor tRNAs and future challenges

Thus far, we have focused on the discussion of RNase P recognition and cleavage of monomeric precursors (*e.g*., *E. coli* pre-tRNA^Tyr^Su3 and model substrates). However, many bacterial tRNA gene transcripts carry more than one tRNA [see *e.g*., (202, 203)]. In *E. coli*, most tRNA genes are clustered in co-transcribed units. Hence, an important task is understanding how RNase P recognizes tRNA transcripts with more than one tRNA sequence. Several ribonucleases in concert with RNase P trim these tRNA precursor transcripts, ultimately generating functional tRNAs. Among these enzymes, endoribonuclease E plays an important role in processing tRNA transcripts by cleaving in the leader and between tRNAs. The cleavage between tRNAs converts the pre-tRNA transcripts into smaller units, which RNase P and exoribonucleases subsequently target to generate functional tRNAs. As discussed above, a number of recent studies in *E. coli* suggest that RNase P is involved not only in generating the matured 5′ end of tRNAs but also plays an important role in separating individual pre-tRNA species from the larger primary transcripts for further processing [for reviews (204, 205), see also (206)].

The spacer regions between two tRNA genes vary in length: in *E. coli*, this distance ranges between 2 and 209 nucleotides (202, 207, 208). As discussed above, the T-even (*e.g*., T4) *E. coli* bacteriophages encode tRNAs. Most of these sequences encode dimeric pre-tRNAs where the individual tRNAs are separated by less than five nucleotides. In the tRNA^Pro^-tRNA^Ser^ dimeric precursor of phage T4, neither tRNA contains the 3′ CCA sequence (Fig. 1*D*), and efficient RNase P processing was shown to prefer CCA at the 3' end of tRNA^Ser^ instead of having UAA (16, 209). The spacer between tRNA^Pro^ and tRNA^Ser^ is only 3 nt. The RPP interacts with residues at positions −8 to −3 in the 5′ leader sequences of monomeric pre-tRNAs, while residues −2 and −1 interact with the RPR [see above (138, 149, 210)]. So, how does bacterial RPP/RNase P interact with the leader given the proximity of tRNA^Pro^ and the tRNA^Ser^ RNase P cleavage site (see Fig. 1*D*)? This question is also relevant to uninfected cells. For example, *E. coli valT* and *lysW*, which are part of the larger "*lysT*" transcript carrying seven tRNAs (211), are only separated by 2 nt (including the 3' ACCA sequence of the upstream tRNA^Val^, *valT*, extends the spacer between *valT* and *lysW* to six nucleotides). The *in vivo* processing by RNase P of the "*lysT*" transcript is initiated by the removal of the ρ-independent transcription terminator located 3′ of the distal tRNA. Subsequently, RNase P processing proceeds in the 3′ to 5′ direction. Similar concerns pertain to other tRNA transcripts, such as those derived from the *valU* and *valV-valW* transcripts (211, 212, 213). *In vitro* data showed conclusively that the 3′ to 5′ processing by RNase P of the *valV-valW* transcript proceeds in a distributive manner (214). In this context, cleavage of a multimeric pre-tRNA by *Bsu* RPR was detected at the predicted sites (63).

It was suggested that *Eco* RPR (M1 RNA) forms dimers to carry out the RNase P reaction (183). Subsequently, it was reported that the *Bsu* RNase P holoenzyme form dimers, and it was discussed that the dimer form binds substrate differently compared to monomeric RNase P (215). The cryo-EM structure of the archaeal *Methanocaldococcus jannaschii* RNase P holoenzyme revealed a dimer (36, 216). Perhaps, dimerization of RNase P might be relevant to the general processing of dimeric and multimeric tRNA transcripts in bacteria. Nevertheless, how bacterial RNase P binds dimeric and multimeric pre-tRNAs is an open and interesting question that remains to be studied in more detail.

RNase P also processes other RNAs, such as pre-tmRNA, pre-4.5S RNA, tRNA-like (pseudo-knot) structures present at the 3′ end of certain plant RNA virus genomes, bacteriophage M3 RNA, bacteriophage-derived antisense C4 RNA, *E. coli* non-coding RNAs transcribed from intergenic regions, transient structures in riboswitches, and mRNAs [(102, 217, 218, 219, 220, 221, 222, 223, 224, 225), for reviews, see refs (54, 226)]. Altman *et al*. were the first to report in 1990 that an mRNA (T4 gene 32) is an RNase P substrate (227). It has recently been suggested that RNase P is involved in mRNA metabolism more broadly (225, 226). These studies are at an early stage and raise many questions such as cleavage site recognition and possible link to ribosome binding and initiation of translation. Interestingly, there is some evidence that the dimeric form of *Bsu* RNase P primarily interacts with the 30S ribosomal subunit, forming an RNase P–30S ribosome complex (228). Also, RNase P was suggested to be associated with RNA degradosome subunits in yeast mitochondria (229). This finding raises the question of whether RNase P interacts with the RNA degradosome in bacteria (230). Ongoing studies in bacteria that investigate the role of RNase P and RNA/mRNA processing using modern technologies such as RNA-Seq will likely provide new and exciting findings that will increase our insight into the biological roles of RNase P (224, 225).

## Gene-targeting technology based on RNase P and M1 RNA: Making use of the unexpected

### Gene-targeting strategy based on RNase P: External guide sequence

**Figure 5:**
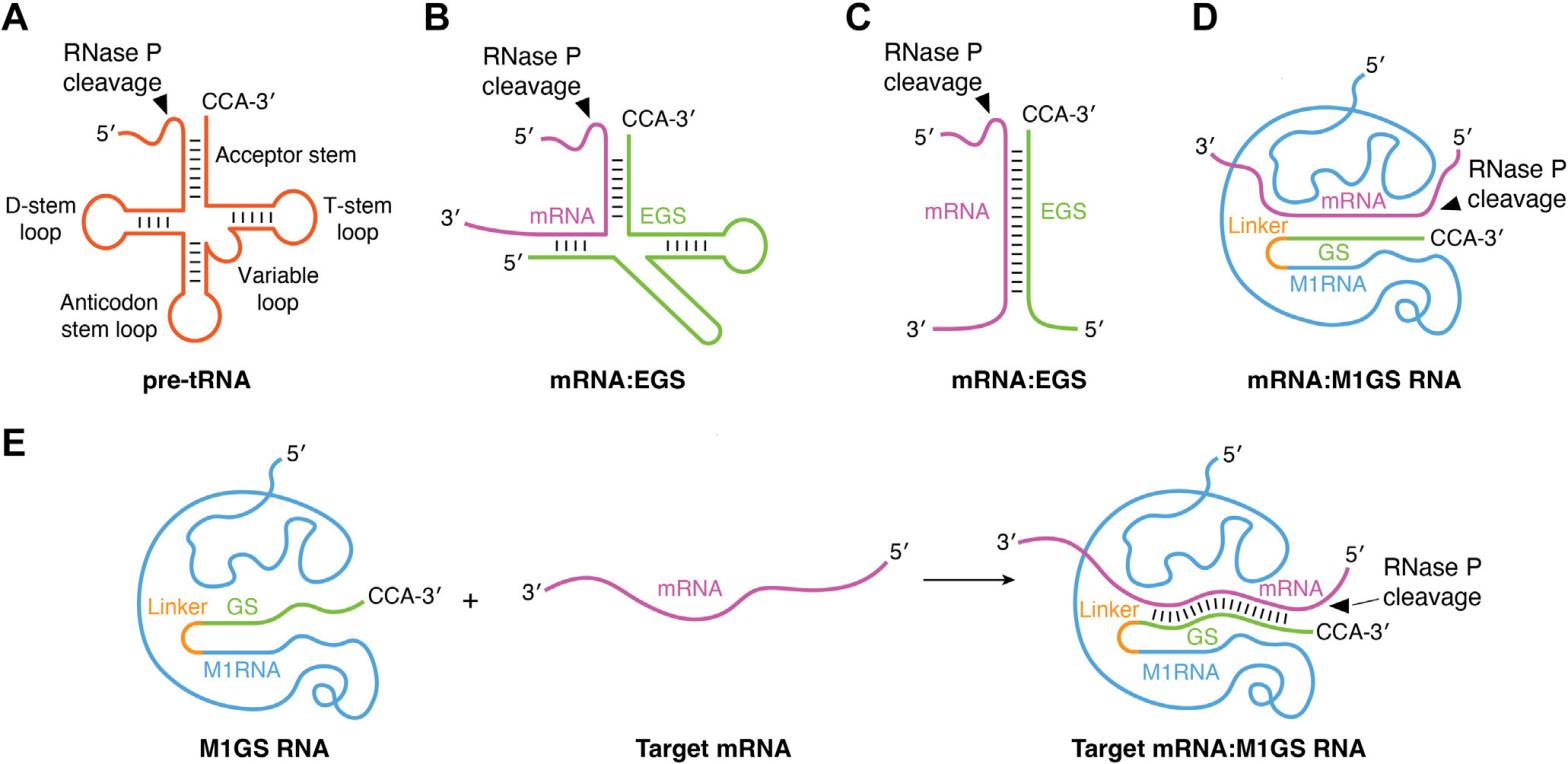
**Representation of various RNase P substrates.** Gene-targeting strategies based on RNase P and M1 RNA with their associated external guide sequences (EGSs) (*A*–*C*). A hybridized complex (*B*) of a target RNA (in *red*, *e.g*. mRNA) and an EGS (in *green*) that resembles a part of the structure of a tRNA structure (*A*) can be cleaved by RNase P and M1 RNA. Substrates in (*A*) and (*B*) can be cleaved by human RNase P and M1 ribozyme. In contrast, the stem structure in (*C*) can only serve as a substrate for M1 RNA and cannot be cleaved by human RNase P. *D*, an M1GS ribozyme–mRNA substrate complex. *E*, binding process of an M1GS ribozyme (in *blue*) with a target mRNA substrate (in *red*). The arrow shows the site of the cleavage by RNase P and M1 RNA.

Altman's early studies on substrate recognition by M1 RNA and RNase P led to the concept of "external guide sequence" (EGS) that could be used for targeted RNA cleavage (190, 231). In this strategy, a custom-designed EGS can guide M1 RNA and RNase P to cleave any mRNA in a sequence-specific manner, provided that the EGS hybridizes to the mRNA and forms a tRNA-like structure [Fig. 5, *A*–*C* (190)]. Two components of an EGS are important for its functions (231). First, the EGS should have a "targeting sequence," which is complementary to the mRNA target and binds to the mRNA substrate through base-pairing interactions. Second, the EGS should also contain an "RNase P–recognized sequence," which resembles a portion of the T-loop and stem and the variable loop and stem of a pre-tRNA. This second sequence enhances the interaction between the EGS and RNase P (and M1 RNA) and is crucial for efficient cleavage of the targeted mRNA by RNase P and M1 RNA (232).

Different designs and constructions have been explored to generate various EGSs for RNase P-EGS applications. For example, Werner *et al*. reported successful construction of short EGSs (233). In the EGS–mRNA complex under this design, the EGS consists of only 15 to 20 nt and resembles the 3′ acceptor stem and 3' TSL regions, and the targeted mRNA resembles the 5′ leader sequence, 5′ acceptor stem, the variable region, and the 5′ TSL of a tRNA (Fig. 5*A*). Another design of EGS links an EGS covalently to M1 RNA, to generate a sequence-specific ribozyme, called M1GS RNA (Fig. 5, *D* and *E*), that can cleave any mRNA substrate that hybridizes with the guide sequence [see below (234)].

### Gene-targeting strategy based on M1GS ribozyme

Investigations by the Altman laboratory showed that M1GS RNA is active and efficient in cleaving numerous mRNAs (234). M1GS RNA is also easy-to-make and could be generated by adding a guide sequence to the 3′ end of M1 RNA (235). The guides should contain a "targeting sequence," which hybridizes to the mRNA target. In addition, the guide sequence should contain an unpaired 3′-NCCA end as present in natural *E. coli* pre-tRNA substrates in order to allow efficient cleavage by the tethered M1 RNA (Fig. 5, *D* and *E*). In the M1GS design, the guide sequence binds to its target mRNA and directs M1 RNA, which is in proximity due to the covalent attachment to the guide sequence, to the cleavage site (Fig. 5*E*). Subsequent studies in the Altman laboratory demonstrated that M1GS RNA can block gene expression in bacteria and virus-infected mammalian cells (Table 1) (234, 236). Further studies showed that M1GS ribozymes cleaved numerous cellular and viral mRNA targets in human cells and in diminishing viral infection in animals (Table 1) [see below and review (235)].

**Table 1:** Representative examples of studies using RNase P ribozymes and the EGS technology against targets associated with human diseases

Target	Method	Effects	Cell/animal model	References
Virus (human influenza virus)	EGS (vector expressed)	Inhibiting viral gene expression and replication	Cultured cells	(249)
Virus (hepatitis B virus)	M1GS and EGS (vector expressed)	Inhibiting viral gene expression and growth and reducing disease progression in animals	Cultured cells and mice	(247, 254, 262)
Virus (HIV)	EGS (vector expressed)	Inhibiting viral gene expression and replication of multiple HIV clades	Cultured cells	(274, 275)
Virus (human cytomegalovirus and herpes simplex virus 1)	M1GS and EGS (vector expressed)	Inhibiting viral gene expression and growth	Cultured cells	(234, 250, 251, 276, 277, 278, 279)
Virus (murine cytomegalovirus)	M1GS and EGS (vector expressed)	Inhibiting viral gene expression and growth and reducing disease progression in animals	Cultured cells and mice	(257, 258, 266, 280)
Virus (Kaposi Sarcoma Associated Herpesvirus)	M1GS (vector expressed) and EGS (chemically synthesized)	Inhibition viral gene expression and growth	Cultured cells	(248, 252)
Bacterium (*Escherichia coli*)	EGS (vector expressed and chemically synthesized)	Blocking bacterial gene expression and growth	Cultured bacteria	(236, 281, 282, 283)
Bacteria (*Salmonella typhimurium*, *Klebsiella pneumoniae*, *Mycobacterium* spp., and *Acinetobacter*)	EGS (chemically synthesized)	Blocking bacterial gene expression and growth	Cultured bacteria	(265, 282, 283, 284, 285)
Bacterium (*Staphylococcus aureus*)	EGS (chemically synthesized)	Blocking bacterial gene expression and growth and reducing disease progression in animals	Cultured bacteria and mice	(282, 284, 285)
Protozoan (*Plasmodium falciparum*)	EGS (chemically synthesized)	Blocking parasitic gene expression and growth	Cultured protozoan	(245, 286)
Human (BCR-ABL oncogene)	M1GS (vector expressed)	Decreasing the target mRNA expression and preventing the function of the BCR-ABL oncogenes	Cultured cells	(263)
Human (PKC and bcl-xL)	EGS (chemically synthesized)	Decreasing the target mRNA expression and blocking the function of the targets	Cultured cells	(264)
Human (CCR5)	M1GS and EGS (vector expressed)	Blocking CCR5 expression and HIV infection	Cultured cells	(287, 288)

In principle, the guide sequence can be tethered to different regions of M1 RNA in addition to the 3′ end of M1 RNA. In their studies with an M1 RNA tethered with a pre-tRNA substrate adjacent to the M1 RNA region for substrate binding, Pace *et al*. demonstrated that an M1 RNA tethered with a 3' region of a tRNA could cleave an RNA substrate resembling the 5′ region of a tRNA including the 5' leader sequence and the 5′ region of the acceptor stem, when the RNA substrate base-paired with the tethered tRNA sequence (237). However, it is unclear whether such customized ribozymes are capable of cleaving mRNA targets and modulating their expression in cultured cells.

The successful use of M1GS ribozyme and the EGSs associated with bacterial and human RNase P in modulating gene expression in different organisms and cells (Table 1) truly reflect Altman's impact and contribution to the development of RNase P as a tool in both basic research and clinical applications.

The RNase P–associated EGS and M1GS ribozymes represent a class of RNA-based gene targeting agents for gene interference and gene-editing applications, which include conventional antisense molecules (238), ribozymes derived from the hammerhead, hairpin, and group I intron ribozymes (239, 240, 241), siRNAs (242), and CRISPR/Cas-associated gRNAs (243). Collectively, these methods lay the foundation for the current widespread use of RNA as a tool to modulate gene expression and as a medicine for clinical applications (244).

### Applications of RNase P as a tool for basic research and for therapy

With the EGS technology, Altman *et al*. successfully modulated the expression of essential genes of several bacteria, including *Salmonella typhimurium*, *Klebsiella pneumoniae, Mycobacterium smegmatis, Staphylococcus aureus*, and *Francisella tularensis* and achieved an antimicrobial effect for the infections of several pathogenic bacteria (Table 1) (245). Furthermore, the RNase P-EGS technology effectively inhibited gene expression and development of *Plasmodium falciparum*, the causative agent of malaria (Table 1) (246). The constructed EGSs appeared to be highly specific and were capable of species-specific targeting.

Altman *et al*. also developed EGSs of different designs using various modifications including morpholino modifiers (245). Furthermore, EGSs could be expressed using expression vectors in bacteria or chemically synthesized and conjugated with a cell-penetrating peptide for direct delivery. If EGS technology can be used in antimicrobial therapy, its ability to achieve species-specific inhibition of bacterial viability could become very useful in circumventing the current limitation of narrow spectrum antimicrobials in inhibiting commensal nonpathogenic bacteria. These results by Altman *et al*. showed the promise of applying EGS technology for antibacterial therapy (245).

Altman's initial work on EGS generated great interest and excitement in developing the EGS technology for antiviral applications (Table 1). His laboratory and numerous laboratories around the world had demonstrated that RNase P and M1GS RNA were effective in blocking infections of HIV, human influenza virus, hepatitis B virus and four herpesviruses including human and murine cytomegalovirus (MCMV), herpes simplex virus 1, and Kaposi's sarcoma-associated herpesvirus (42, 235, 247, 248).

Among the first antiviral EGS studies, Plehn-Dujowich *et al*. constructed EGSs against the mRNAs coding for the nucleocapsid protein and polymerase of human influenza virus (249). They further showed that targeting two different mRNAs simultaneously by the EGS technology appeared to reduce viral growth more than targeting of a single mRNA. In their EGS studies against HIV, which infects CD4 T cells and decreases their levels *in vivo*, Hnatyszyn *et al*. showed that RNase P effectively blocked HIV gene expression and replication (275).

Liu *et al*. showed that RNase P–associated EGS RNAs and M1GS ribozymes were also highly active in targeting the mRNAs of herpes simplex virus 1 and human cytomegalovirus *in vitro* and blocking the gene expression and replication of these viruses in cultured cells (250, 251). Furthermore, they demonstrated that exogenous administration of chemically synthesized 2′-*O*-methyl-modified EGS to Kaposi's sarcoma-associated herpesvirus–infected human primary-effusion lymphoma cells significantly inhibited viral expression and growth (252).

Studies were also carried out to investigate the activity of EGSs and M1GS ribozymes for modulating gene expression and blocking viral infection in mice (Table 1). In one study, M1GS ribozyme-expression plasmid constructs were delivered in mice using a hydrodynamic transfection procedure (253). Expression of M1GS ribozymes were found in the spleens and livers and blocked gene expression and infection of MCMV *in vivo*. The delivery of M1GS expression constructs led to the inhibition of MCMV pathogenesis and prolonged the survival of the infected mice (253). In another study, the EGS expression constructs were delivered using a *Salmonella*-based vector into mice (254). The delivery of the EGS expression constructs effectively blocked the gene expression and replication of hepatitis B virus (HBV) *in vivo* (254).

To develop better EGSs, Yuan and Altman employed *in vitro* selection procedures for generating variant EGSs that were more efficient in inducing human RNase P cleavage of a target mRNA than the EGS derived from a natural tRNA sequence (232). Similarly, to further enhance the efficiency of ribozymes, *in vitro* selection procedures were used to select for M1GS variants that efficiently cleaved an mRNA (255). These efforts led to the development of numerous M1GS ribozyme variants that cleave their mRNA substrates more efficiently than the M1GS ribozyme derived from the M1 RNA sequence (235, 256). Importantly, when expressed in cultured cells and in mice, the EGS and M1 ribozyme variants selected *in vitro* were more effective in blocking viral gene expression and infection than the EGS derived from a natural tRNA sequence and the M1GS ribozyme derived from the WT M1 RNA sequence, respectively (255, 257, 258, 259, 260, 261, 262).

In using the EGS technology in anti-tumor applications, Sánchez-García and colleagues constructed M1GS ribozymes to hydrolyze chimeric RNAs originating from chromosomal abnormalities (263). M1GS RNAs appeared to be highly specific and only cleaved the target chimeric mRNA *in vitro*. Furthermore, expression of the constructed RNase P ribozymes inhibited the oncogenic effect of BCR-ABL function in cultured mammalian cells (263).

In another study, Stein et al. generated EGSs to induce RNase P–mediated cleavage of the mRNA that encodes protein kinase C-α and antiapoptotic protein bcl-xL (264). They administered chemically synthesized 2′-O-methyl–modified EGSs into T24 bladder carcinoma cells for specific downregulation of protein kinase C-α and bcl-xL expression. They did not observe any nonspecific cleavage, which is usually associated with RNase H–based methods (264). These experiments provided direct evidence that RNase P–mediated cleavage induced by EGS is highly specific in targeting its mRNA. Collectively, these results suggested a general applicability of the EGS technology for anticancer applications (264).

Highly active RNase P ribozymes and EGSs were generated using *in vitro* evolution approaches (42, 235). Some of these molecules were highly effective in blocking gene expression in cultured cells and in mice. Biochemical characterization suggested that the mutations found in the selected ribozyme variants enhance the rate of cleavage and improve binding to specific mRNA regions, which are not present in pre-tRNAs (251, 255). Similar studies showed that the selected EGSs increased their targeting activity by increasing tertiary interactions affecting folding of the mRNA–EGS complex into a tRNA-like structure in addition to enhancing the interactions of the EGSs with RNase P (236, 256). These promising results have laid the foundation for developing better and more active RNase P ribozymes and EGSs for gene-targeting applications.

### Advantage and disadvantage of the RNase P ribozyme and EGS technology

Traditional antisense technology employs cellular RNase H to degrade the mRNA target (238). However, nonspecific cleavage at non-targeted sites is a potential problem, as RNase H does not require a 100% complementary duplex for direct cleavage of the target mRNA (238). Compared to conventional antisense DNA and RNA, M1GS ribozyme can be highly specific in cleaving its targeted mRNA (235, 263). For example, Sanchez-Garcia et al. showed that M1GS ribozyme can be specific in cleaving one substrate over another even though the two substrates share the first nine contiguous base pairs complementary to the guide sequence (263). In another study using short EGSs complementary to their target sequences to induce endogenous RNase P holoenzyme to cleave their targets and reduce bacterial viability in *E. coli*, three nucleotides unpaired out of a 15-mer EGS still favor complete inhibition of bacterial viability by the EGS but five unpaired nucleotides do not (265, 281, 282, 283, 284). These interesting observations suggested that the targeting specificity of M1GS ribozyme may not be the same as that of the EGS when it is separated from M1 RNA or the holoenzyme *in vitro*. Furthermore, they implied that the sequence specificity of M1GS ribozymes in the presence of various proteins in human cells is perhaps different from that of the EGS interacting the bacterial holoenzyme and other proteins in *E. coli*. Additional studies on these issues, especially the specificity of M1GS ribozymes and EGS coupled with RNase P in human cells, should provide insight into the mechanism of how they achieve sequence specificity for targeting and facilitate the development of highly specific EGSs and M1GS ribozymes for therapeutic applications.

Compared to other ribozymes including hammerhead and hairpin ribozymes, M1GS ribozyme possesses several unique features as a gene-targeting tool. First, M1GS ribozyme can fold into a defined active conformation in the absence of its substrates. Second, while M1GS can cleave any designed sequence, hammerhead and hairpin ribozymes are limited by the requirement for the presence of specific nucleotide sequence (–GUX–) in the target mRNA for the cleavage to occur (239, 240, 241). Furthermore, a single point mutation in the required GUX sequence could render the ribozymes ineffective for target mRNA cleavage. The low specific sequence requirements at the cleavage site provide M1GS ribozyme with better flexibility to be used against almost any target, including positionally fixed target sites such as the fusion junction of two chromosomes resulting in an oncogenic chimeric mRNA (263). Third, the small ribozymes may have the disadvantage to be either rather inefficient under physiological conditions (*e.g*., in the case of the minimal hammerhead ribozymes) or to catalyze ligation quite efficiently (*e.g*., in the case of natural hammerhead ribozymes or the hairpin ribozymes); ligation will limit the efficiency of target cleavage, a clear disadvantage relative to RNase P and M1GS RNAs, which do not catalyze the reverse reaction.

Compared to other nucleic acid–based gene interference approaches, the EGS technology with the use of endogenous human RNase P exhibits several unique and attractive features as a gene-targeting tool. First, the mechanism of the EGS technology is different from other nucleic acid–based gene-targeting approaches in degrading the target mRNA. The EGS technology uses endogenous RNase P, which is one of the most ubiquitous, stable, and efficient enzymes in all types of cells (42, 245). This essential enzyme is highly expressed and is responsible for the processing of all tRNA precursors that account for approximately 2% of total cellular RNA. The action of RNase P in the presence of the EGS will result in irreversible cleavage of the target mRNA in a highly efficient catalytic fashion.

Second, the sequence specificity of the EGS technology is governed by two different types of interactions between the EGS and the target mRNA: (i) the base-pairing interactions in which the sequence of 12 nt in the EGS hybridizes with the target mRNA and (ii) the interactions between the target mRNA and the other part of the EGS sequence (equivalent to the T-stem and T-loop and variable regions of a tRNA) which are required for folding of the RNase P–recognizable tertiary structure (235). Thus, the EGS-based technology is highly specific and does not generate nonspecific "irrelevant cleavage" that is observed in RNase H–mediated cleavage induced by conventional antisense phosphorothioate molecules (252, 266). Third, cells expressing these molecules for more than 40 days appear to be normal indicating that EGSs exhibit little sign of cytotoxicity (250, 251, 252, 264).

In recent years, the use of the RNA interference (RNAi) approach to degrade mRNA associated with human diseases has been the focus for nucleic acid–based gene interference studies and several compounds based on RNAi have been approved for clinical therapy against specific human diseases (242). RNAi has the advantage of utilizing the cellular machinery in its process to knockdown mRNA and can be effective in small concentration. However, the siRNA technology may "sequester or misguide" a cellular machinery which may have consequences for cell function not foreseeable at present. More recently, genome-editing approaches such as those with CRISPR/Cas-associated gRNAs (243) have shown promising results for potential clinical applications.

Studies comparing the effects of the RNase P–based gene-targeting approach with those of other RNA-based methods have not been extensively performed. Results from Hayday *et al*. showed that shRNA and native tRNA-derived EGSs could both target the thymosin beta gene in cultured cells, but the extent of RNA reduction with shRNA was significantly greater (266, 267). More studies are needed to compare the activity and effectiveness of M1GS RNA/the EGS technology and RNAi and CRISPR-Cas–based approaches for modulating gene expression in human cells.

As with any gene therapy design, stability and delivery of the agents remain a big concern. The delivery problem affects the siRNA technology to the same extent as the EGS technology. For stability, the ribozymes and EGSs could be chemically synthesized with 2′ hydroxyl modification and/or phosphorothioates to resist cellular endonucleases (244). As an alternative to the viral vector approach, smaller ribozymes can be delivered *ex vivo* by encapsulating them in liposomes or other biodegradable polymeric matrix (244, 252, 264). Endogenous and stable expression of M1GS ribozyme by viral vectors remains one of the most practical choices for M1GS expression and delivery. EGSs are small molecules of 25 to 60 nucleotides. Therefore, the EGSs can be easily synthesized and modified chemically (244, 252, 264). Thus, an EGS can be delivered directly (in naked form or with the aid of liposomes) to cells as well as delivered by expression vectors such as retroviral vectors.

### Future directions and challenges

RNase P–associated EGS and M1GS ribozyme represent promising gene-targeting agents for both basic research and clinical applications. They are unique due to the use of RNase P and its catalytic RNA. Thus, the RNase P–based technology can complement other RNA-based gene-targeting approaches including those with conventional antisense molecules, ribozymes derived from the hammerhead, hairpin, and group I-intron ribozymes, RNA interference, and CRISPR/Cas gene editing methods. Future studies may be needed to address several challenges and develop these agents with the following considerations. The cleavage efficiency and specificity of the RNase P guide sequence technology *in vivo* will be further improved by better design and construction of EGSs and M1GS ribozymes including those selected *in vitro*. Moreover, the delivery and expression of the EGS and M1GS ribozymes can be optimized with the recently developed novel vectors and lipid carrier methods that have been shown to be successful for clinical applications (244, 268). These studies will facilitate the development of the RNase P guide sequence technology in clinics for treatment of various human diseases including infections and cancers.

## Conflict of interest

The authors declare that they have no conflicts of interest with the contents of this article.
